# Effects of Pygidial Secretion (Zoopesticide) on Histopathological Changes in the Male Accessory Reproductive Glands of Adult Male Insect *Odontopus varicornis* in Relation to Reproduction

**DOI:** 10.4103/0971-6580.68345

**Published:** 2010

**Authors:** M. Lousia, Selvisabhanayakam, V. Mathivanan

**Affiliations:** Department of Zoology, Annamalai University, Annamalai Nagar, Cuddalore District, Tamil Nadu, India

**Keywords:** Male accessory reproductive glands, *Odontopus varicornis*, pygidial secretion

## Abstract

Indiscriminate use of pesticides for the eradication of pests causes tremendous changes to the environment and also to other nontarget organisms. To prevent such contamination of the environment and save nontarget species, zoopesticides are increasingly used as they are cost effective, eco-friendly, safe, and sustainable in the field of agriculture. The present study was undertaken to find out the effect of pygidial secretion (zoopesticide) on *Odontopus varicornis*. The insects were exposed to pygidial secretion for 24, 48, 72, and 96 hours and its sublethal concentration was found to be about 2.8% for 48 hours. When the insects were injected with sublethal concentration 2.8% for 48 hours, the study revealed certain remarkable changes in the histopathology of the male accessory reproductive glands (MARGs) such as disintegration of epithelial cell wall, swollen nucleus, vacuolization of cytoplasm, highly pycnotic and necrotic epithelium, enlargement of epithelial cells, and disorganized tissues. It is suggested that zoopesticide causes several histopathological damages in the MARGs of *O. varicornis* and affects the reproductive potentiality of *O. varicornis*.

## INTRODUCTION

Man and insects have been at war for the same food and same places to live in. Insects attack man and his domestic animals by causing diseases, they destroy his property and crops, and hence insects are of very great importance to man. Reproductive physiology of male insect is a complicated phenomenon, and it deals with the structure and functions of various tissue components of the system. Male accessory gland products have attained great importance in insect reproduction as they are a means of transport for sperm and can form a mating plug. They have specific compounds that can modify the behavior and physiology of mated females.[1] The principal secretory products of these glands in male insects are proteins, carbohydraes, and lipids.[2,3] The pygidial secretions are safe, nontoxic, nonpolluting, and avoid accumulation of residual effects on agricultural products.

Pygidial gland opening in the pygidium or posterior tip of the abdomen of insects are called pygidial glands. They may secrete poisonous or abnoxious substance as a protection for the insect. Those glands in Carabidae, for instance, secrete butyric acid.[4] The most pygidial gland compounds in Dytiscidae are aromatic aldehydes (e.g., hydroxyl benzaldehyde), esters (e.g., methyl *p*-hydroxy benzoate), and acids (e.g., benzoic acid). Carabides also produce a diverse array of pygidial chemicals. These are generally hydrocarbons, aliphatic ketones, saturated esters, formic acid, higher saturated acids, unsaturated aliphatic acids, phenols, aromatic aldehydes, or quinines.[5]

Insects have evolved a multitude of chemical and behavioral changes against other offensive organisms. The defensive secretion of insects and other terrestrial arthropods have been investigated ever, since formic acid was isolated and identified from ants.[6] Many arthropods produce secretions which are used for the defense and offence against other arthropods of small predatory vertebrates or may be toxic to plants.[7] These defensive secretions are ejected as drops or get spread over the body surface of the insect or sprayed at or injected into the body of the animal. They are clearly formidable poisons, capable of introducing considerable toxic effects, with a broad rather than narrow spectrum having repellence. The natural products of zoopesticides can be used for the purpose of controlling insect pests and also have a number of advantages over the conventional chemical insecticides. Hence, the present study was undertaken to find out the impact of pygidial secretion on the test insect, *O. varicornis*.

## MATERIALS AND METHODS

The insects collected from the fields and gardens were reared in wooden cage, each measuring about 30 cm × 22 cm × 28 cm at the laboratory temperature of 28±2°C and relative humidity of 80% ± 5%. The insects were fed daily with soaked cotton seeds (*Bombax ceiba*) as well as with seeds of its higher plant, *Stericulia foetida* and *Gossypium* sp. An additional food of the pieces of chow-chow (*Sechium edule*) was also given to these insects.

### Histological technique

Male insects of *Odontopus varicornis* were selected from the cage. The adult insects were dissected out using insect Ringer solution.[8] The removed tissues were fixed in aqueous Bouin’s fixative. After 24 hours of fixation, the tissues were processed for dehydration using ascending grades of alcohol according to Gurr.[9] The tissue was gross stained in 70% aqueous eosin to facilitate orientation during embedding. The tissues after dehydration in absolute alcohol and acetone were cleared in xylol and finally embedded in paraffin wax (58–62°C). Sections cut at 6-μm thickness were deparaffinized using descending grades of alcohol, stained with hematoxylin, counter-stained with aqueous eosin for microscopical observations, and microphotographs were taken.

## RESULTS AND DISCUSSION

**Figure 1:**
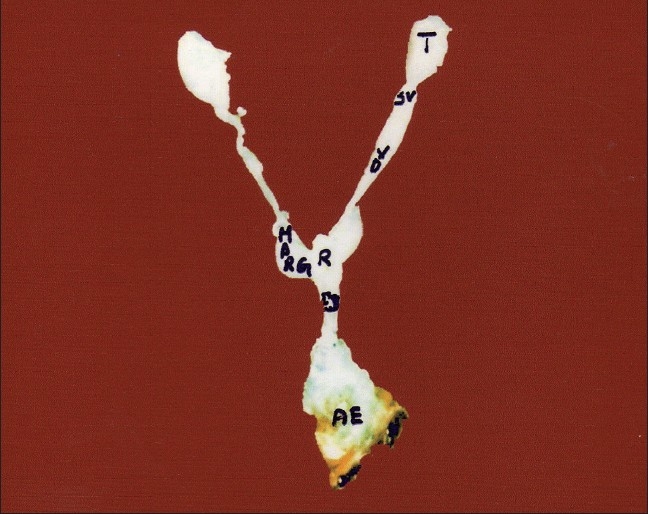
Morphology of male reproductive system; AE - Aedeagus; Ej - Ejaculatory duct; MARGs - Male accessory reproductive glands; R - Reservoir; SV - Seminal vesicle; T - Testis; VD - Vas deferens

The accessory glands appeared as transparent, short, pear-shaped body, pale white in color, and richly supplied with tracheal tubes. They lie at the posterior median end of the abdominal cavity just below the junction of vasa-deferentia, and the pear-shaped structures situated on either side of the reservoir which led to the common ejaculatory duct (Ej) [Figure 1]. These glands were mesodermal origin in *O. varicornis*; hence they are called “mesadene glands.” The glands showed periodic cyclicity, i.e., the size varied depending upon the activity of the gland and age of the insect. These glands constitute a continuous tube, curved and coiled without any order to form a globular mass of tube, ensheathed by thick cuboidal cells of single layer. Superficially, they look like a wrinkled ball and were colorless. These glands were also called mucus glands or cement glands. The secretions of these glands were poured into the posterior end of the ampulla [Figure 1]

### Histology of male accessory reproductive glands

**Figure 2:**
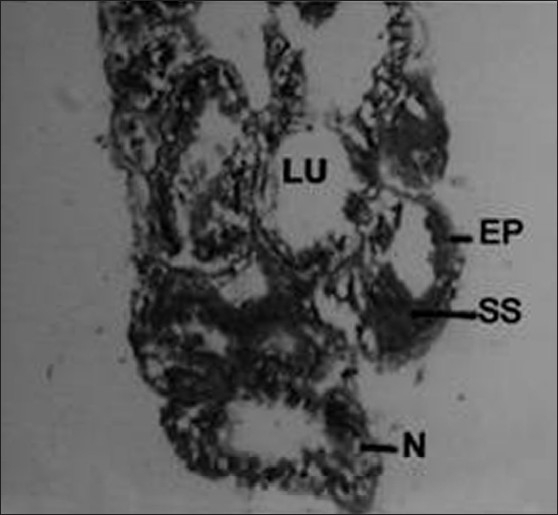
Cross section of male accessory reproductive gland (MARGs) of control insect (Xca100); EP - Epithelium; LU - Lumen; N - Nucleus; SS - Secretory substances

**Figure 3:**
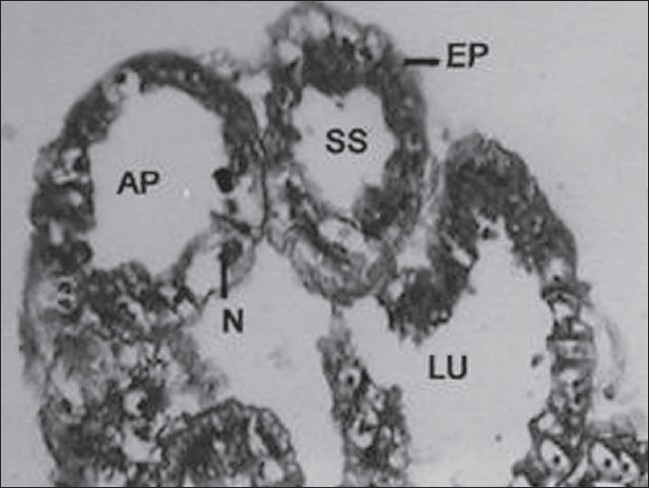
Cross section of male accessory reproductive gland (MARGs) of control insect (Xca400); EP - Epithelium; LU - Lumen; N - Nucleus; SS - Secretory substances; AP - Apocrine mode of secretion

**Figure 4:**
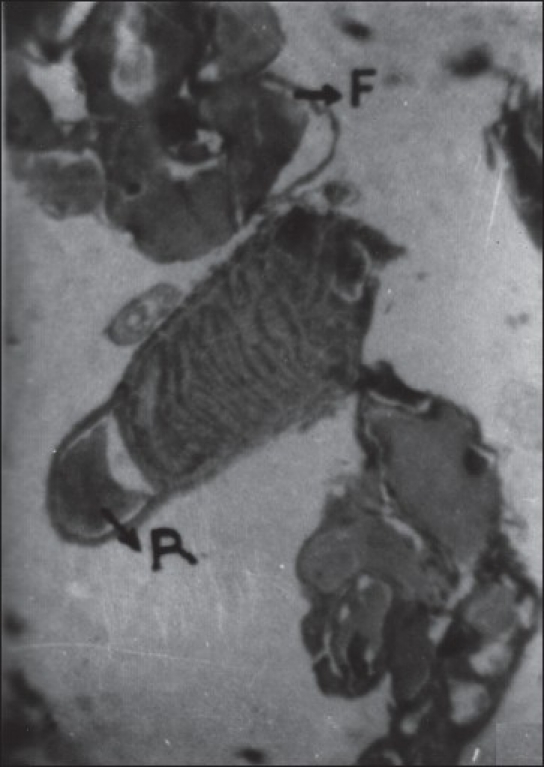
Cross section of (MARGs) a pair of male accessory reproductive gland and a central reservoir. (Xca100); F - MARG follicle; R - Reservoir

The accessory glands in male insect were made up of long continuous glandular tube, curved, coiled to form a compact globular mass. A number of lobules appeared in sections, which were lined with glandular cells supported by a basement membrane [Figure 2]. These cells were columnar to cuboidal with oval nuclei and granular cytoplasm. The cytoplasm contained vacuoles with secretions, which was discharged periodically into the distended lumen of the lobule [Figure 3]. The short and pear-shaped accessory reproductive gland in histological sections seems to be composed of 10–12 follicles. Each follicle of this gland was simple epithelial tubular structure without distinct cell boundaries [Figure 4]. It was formed by a single layer of epithelium, which enclosed a central lumen. The epithelium was surrounded by a thin muscular layer. The diameter of the lumen was not uniform in different regions of the follicle.

**Figure 5:**
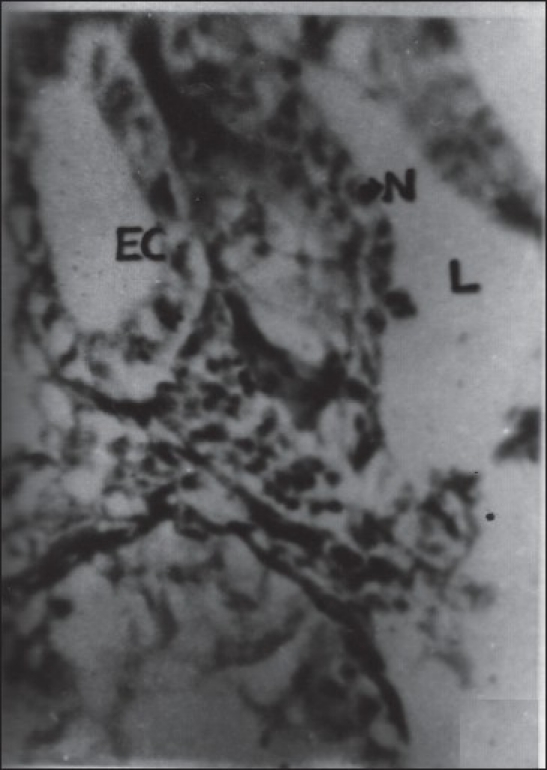
Cross section of the (MARGs) showing columnar epithelial organization of the gland. (Xca400); EC - Epithelial cell; N - Nucleus; L - Lumen showing without secretory material

**Figure 6:**
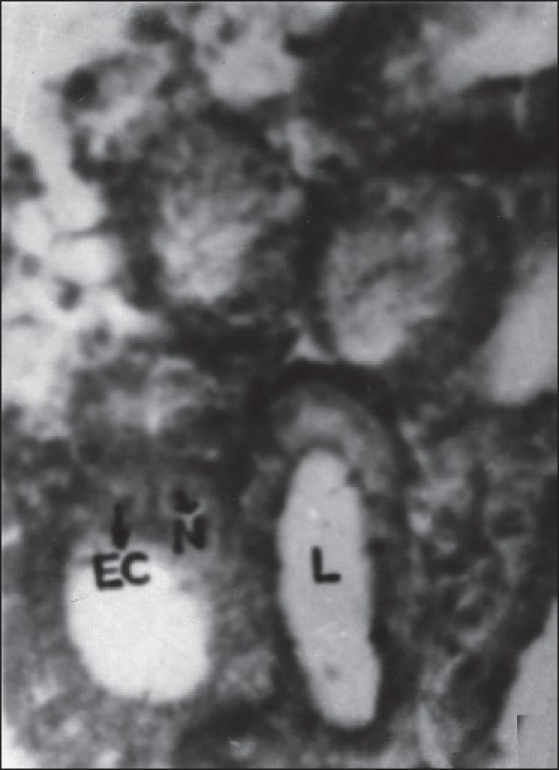
Cross section of the male accessory reproductive gland (MARGs) showing epithelial organization of the gland. (Xca400); ECEpithelial cell; N- Nucleus; L-lumen showing without secretory material

**Figure 7:**
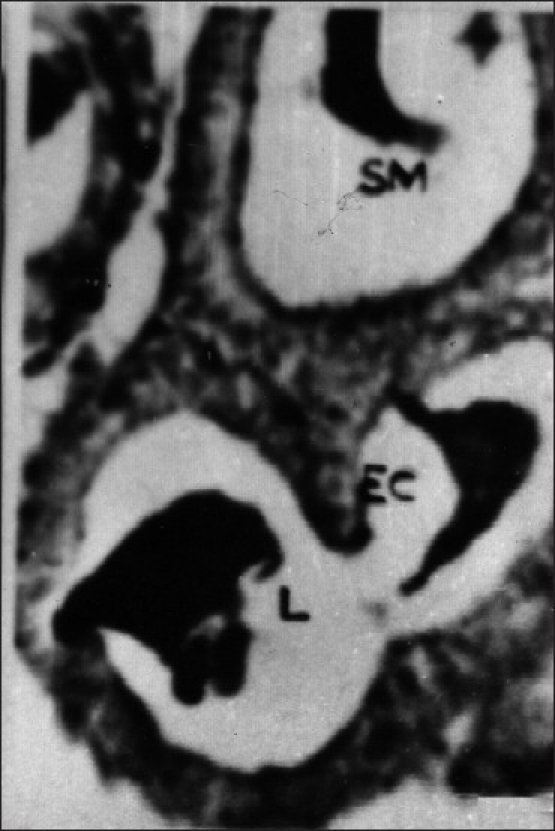
Cross section of the male accessory reproductive gland (MARGs) showing secretory material with lumen. (Xca400); EC- Epithelial cell; N- Nucleus; L-lumen showing without secretory material; SM- secretory material

The epithelial layer was composed of columnar cells each with a large spherical or oval nucleus. The chromatin granules were intensely stained with hematoxylin and densely packed. The secretion of these cells appeared in the form of fine eosinophilic granules, which were released into the lumen by the rupture of the cell membrane and thus it represents the apocrine mode of secretion. The secretion was discharged into the reservoir (ampulla) and then into the Ej. There was no evidence for the formation of spermatophore in this insect. The spherical or oval-shaped nucleus was located in the center of the epithelial cell. The chromatin materials were found to be densely packed in the nucleus and the nucleolus was not discernible [Figures 5 and 6]. The lumen of the gland is filled with a higher quantity of granular secretary materials in control insects [Figure 7].

According to Lepold,[10] the fluid mixture of secretions has certain components, which may promote sperm maturation and provide nourishment for stored sperms. The activation of sperm is one of the functions of this gland.[11] Baskar[12] and Selvisabhanayakam[2] have shown that the secretion of this gland is utilized as an energy precursor for the elaboration of seminal fluid from male to female during mating, as evident by the occurrence of reduced quantity of secretion after mating in *Serinetha auger* and *O. varicornis*. Similar findings have also been reported by Padmanabhan[13] in *Aspongopus Janus*. The secretary material of this gland is used for the formation of the wall of the spermatophore, activation, nourishment, and motility of sperms.

Leahy[14] has reported that the secretions of accessory gland in *Schistocerca gregaria* do influence both maturation and release of eggs in females. Lum[15] has reported that the secretions of this gland are used as a medium for spermatozoa during ejaculation in *Aedes aegypti*. In *Apis mellifera*, the mating plug is synthesized by this gland. In the present study, it has been shown that the secretion of male accessory reproductive glands (MARGs) of control insect was utilized as an energy source for the elaboration of seminal fluid from male to female during mating as evidenced by the occurrence of reduced quantity of secretion, and also the glands were covered with a thin outerbasement membrane and an innerlumen beset with less secretary substances.

### Histopathology of MARGs

**Figure 8:**
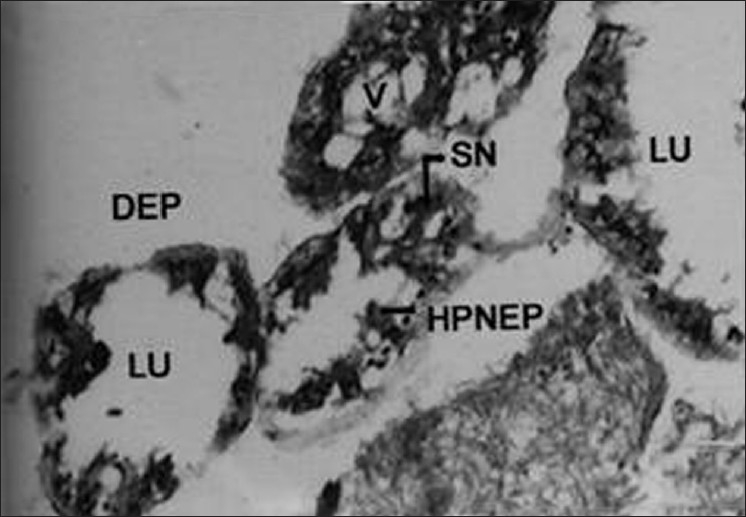
Cross section of male accessory reproductive gland (MARGs) of treated insect (Xca100); SN - Swollen nucleus; LU - Lumen; DEP - Disintegrated Epithelium; HPNEP - Highly pycnotic necrotic epithelium; V - Vacuole

**Figure 9:**
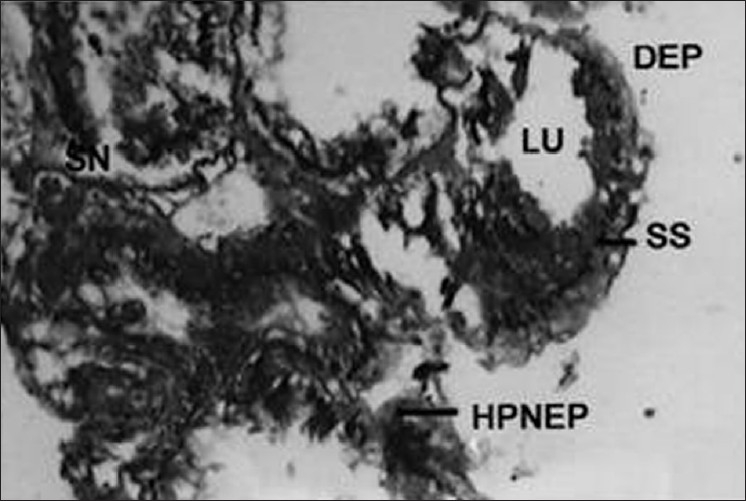
Cross section of male accessory reproductive gland (MARGs) of treated insect (Xca400); SN - Swollen nucleus; LU - Lumen; DEP - Disintegrated Epithelium; HPNEP- Highly pycnotic necrotic epithelium; SS - Secretory substances

Generally, the accessory glands were enlarged; in certain cases, only one gland was enlarged while the other remained small. Glandular layers varied in structure, they were either very thick or thin with large lumen [Figure 8]. The secretions were generally found to be nonhomogenous and were found more toward one side of the lumen. Disintegration of epithelial cell walls, enlarged nuclei, vacuolization of cytoplasm, enlargement of epithelial cells, and nuclear pycnosis of cells were observed [Figure 9] when insects were intoxicated with the zoopesticide pygidial secretion.

Similar findings have been reported by many of the workers with chemical as well as phytopesticides. Hopp[16] has reported that the direct application of chlorobenzene to *Pediculus humanus* has been reported lysis of cells and nuclei of epidermis, breakage of mitotic divisions. Jayakumar[17] has observed the same changes in *O. varicornis* treated with dimethoate.

Glandular layers of the accessory glands varied in structure, and secretions were found to be nonhomogenous in the dimilin-affected *Dysdercus similis* by Arunakumari.[18] Similarly, Sita[19] has reported in steroid-treated D. *similis* that accessory glands were deformed with reduced lumen and reduced secretions. In diflubenzuron- and fenvalerate-treated *Cylas talis formicarius*, accessory glands were reduced. The mesadene glands and bulbus ejaculatories appeared to have lost their definite indentify in *Leptocoris coimbatorensis* and *Dysdercus cingulatus* when treated by renfluron as reported by Sathyanarayana.[20] Balakrishnan[21] has noticed several histopathological changes in *Pherosphus lissoderus* treated with dimethoate, which are similar to the results of other workers namely Nirmaladevi[22] for *Catacanthus incarnates* exposed to phosphomedon, Thiruvasagam[23] for *A. janus* exposed to nimbicidine, and Palanisamy[24] for *Laccotrephes ruber* exposed to mercuric chloride. Sumathi[25] has reported that endosulfan affected the degenerative changes in accessory reproductive organs of *Gryllotalpa africana*. Rajathi[26] exposed to heavy metal mercury for *Spherodema rusticum* and also Rameshkumar[27] for *Laccotrephes ruber* exposed to heavy metal zinc.

In the present study, it has been observed that the reduction in the size, structure of accessory gland, disintegration of cell walls, pycnotic nuclei, reduction in glandular secretion, and vacuolized cytoplasm were attributed due to the defensive secretion (zoopesticide) that brought about reproductive disturbances and sterility in the test insect, *O. varicornis* than the control insects.
